# Double anévrisme Sylvio-mésentérique révélant une endocardite infectieuse

**DOI:** 10.11604/pamj.2016.25.103.9911

**Published:** 2016-10-20

**Authors:** Amine Ghalem, Houssam Laachach, Alaa Fliti, Abdelmalek Elyandouzi, Abdelwahab Elkasimi, Nabila Ismaili, Noha El Ouafi

**Affiliations:** 1 Service de Cardiologie, CHU Mohammed VI, Oujda, Maroc

**Keywords:** Endocardite infectieuse, complications, anévrismemycotique, Infective endocarditis, complications, mycotic aneurysm

## Abstract

L'endocardite infectieuse est une urgence qui est diagnostiquée classiquement dans le cadre d'un syndrome infectieux associé à un souffle auscultatoire cardiaque. Elle peut mettre en jeu le pronostic vital via ses complications. Nous rapportons le cas d'une endocardite révélée suite à des manifestations neuro-abdominalesliées à un double anévrisme mycotiquesylvio-mésenterique et qui a bien évolué sous traitement medico-chirurgical.

## Abstract (en)

Infective endocarditis is an emergency condition usually diagnosed in the context of an infectious syndrome associated with heart murmur. It can lead to life-threatening complications. We report the case of a patient with endocarditis presenting as neuro-abdominal manifestations associated with sylvian and mesenteric arteries mycotic aneurysm. Evolution was favorable under medical-surgical treatment.

## Introduction

L'endocardite infectieuse (EI) est une affection rare et grave du fait de ses complications. L'anévrisme mycotique qui complique 2,5 à 10 % des cas d'EI en représente une importante cause de morbidité et de mortalité en dépit de l'antibiothérapie [1]. Nous rapportons le cas d'un patient de 30 ans, admis pour douleurs abdominales et hémiparésie avec fièvre en rapport avec un anévrisme mycotique de l'artère mésentérique supérieure et de l'artère sylvienne profonde compliquant une endocardite infectieuse à localisation mitrale.

## Patient et observation

**Figure 1:**
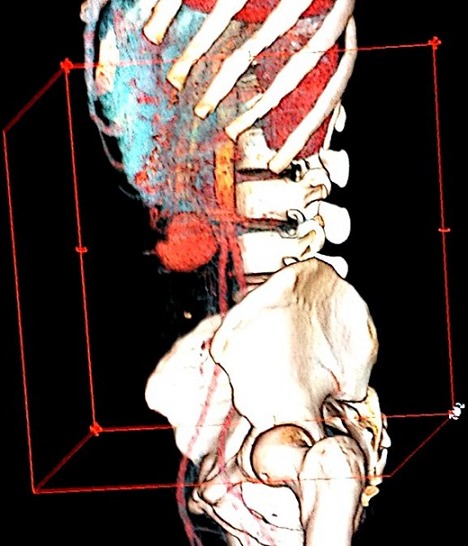
Aspect anévrysmalmycotique de l’artère mésentérique supérieure vu sur une reconstruction scannographique

**Figure 2:**
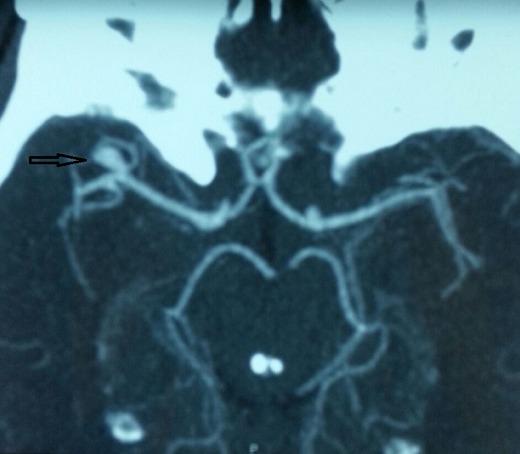
Coupe TDM d’angioscanner objectivant un anévrysme sylvien antérieur droit

**Figure 3:**
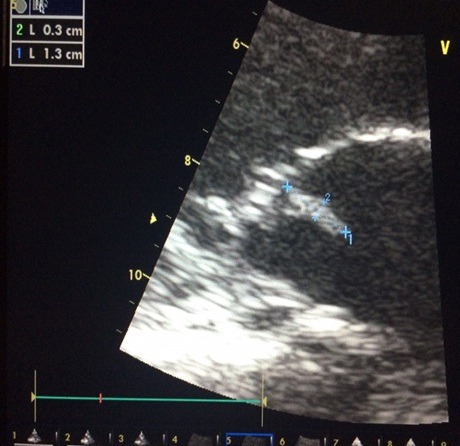
Aspect échocardiographique d’une végétation de la GVM

Il s'agit d'un patient âgé de 30 ans, tabagique chronique à raison de 8 paquets année, admis pour une fièvre chronique évoluant depuis 2 mois avec notion de prise d'une antibiothérapie durant 07 jours arrêtée il y a 08 jours, associée à des douleurs abdominales et une lourdeur de l'hémicorps gauche d'installation récente. L'examen physique trouvait un patient conscient, (apyrétique) fébrile à 38,3^®^, stable sur le plan hémodynamique, avec des râles crépitant en basithoracique droit, un souffle d'insuffisance mitrale 5/6, une hémiparésie gauche et une sensibilité épigastrique. Une échographie abdominale objectivait une lésion d'aspect anévrismal accolée à l'aorte abdominale. Un complément TDM Cérébral et thoraco abdomino pelvien montrait un volumineux anévrisme mycotique du segment distal de l'artère mésentérique supérieur (AMS) associé à un épaississement pariétal et un infarctus splénique et rénal bilatéral, et la reconstructionscannographique 3D de la région abdominale a mis clairement en relief la lésion anévrismale mésentérique (Figure 1). Par ailleurs, à l'étage cérébral, est objectivé un anévrisme mycotique d'une branche de l'artère sylvienne (Figure 2) avec hypodensité pariétale adjacente évoquant une zone d'ischémie. Devant la fièvre chronique, l'AVC ischémique, et l'anévrisme mycotique de l'AMS, une ETT était réalisée montrant une végétation mobile appendue à la GVM mesurant 18 mm de diamètre avec IM sévère et VG dilaté de bonne fonction systolique (Figure 3). Deux séries de 03 hémocultures sont revenues négatives, le fond d'œil n'a pas objectivé d'anomalies. Le diagnostic d'endocardite infectieuse à localisation mitrale, à hémocultures négatives (probablement décapitées) et multi-compliquée était retenu (un critère majeur + 3 critères mineurs). Le traitement était basé initialement sur une bi-antibiothérapie (Amoxicilline + Gentamicine) intraveineuse avec cure chirurgical urgente de l'anévrisme de l'artère mésentérique supérieure par une mise à plat vu le risque potentiel de sa rupture. Les suites opératoires étaient simples. L'évolution était marquée par la régression du syndrome inflammatoire et de l'anévrisme de l'artère sylvienne. Le patient a été adressé ensuite pour remplacement valvulaire mitral associé à une annuloplastie aboutissant à une bonne évolution ultérieure.

## Discussion

L'endocardite infectieuse (EI) est une affection rare, d'expression polymorphe, et grave du fait de ses complications. Le ***staphylococcus et le streptococcus*** sont les organismes les plus communément isolés. L'anévrysme mycotique, qui complique 2,5 à 10 % des cas d'EI, en représente une importante cause de morbidité et de mortalité en dépit de l'antibiothérapie. Ces anévrysmes peuvent se développer aux dépens de nombreuses artères : l'aorte, les artères cérébrales, viscérales et périphériques. Les localisations viscérales sont dominées par l'atteinte de l'artère splénique qui représente 60 % des cas, suivie de celles de l'artère hépatique (20 %) puis de l'artère mésentérique supérieure (AMS) (environ 5,5 %) [1–3] avec rarement une localisation multiple [4–6]. Les anévrysmes mycotiques représentent 50 à 60 % des anévrysmes de l'AMS, principalement chez les sujets âgés de moins de 50 ans à la suite d'une endocardite bactérienne subaigüe comme c'était le cas dans notre observation. Leur risque de rupture est important (entre 38 et 50 %) avec un taux élevé de mortalité (entre 40 et 60 %) [4]. Les anévrysmes mycotiques viscérales sont souvent diagnostiqués tardivement du fait de leur caractère asymptomatique, et peuvent évoluer vers la rupture dont l'issue peut être fatale. L'angioscanner est l'examen le plus utile pour objectiver les anévrysmes aortiques infectieux, ainsi que ceux cérébraux, possédant en outre l'avantage d'être plus disponible que l'angio-IRM (imagerie par résonance magnétique) [1–4]. En imagerie, ils sont caractérisés par leur croissance rapide, une absence de calcification de la coque, leur aspect multilobé, Leur forme sacculaire et une infiltration des tissus mous [5]. Le traitement classique des anévrysmes mycotiques de l'AMS est médical (bi-antibiothérapie) et chirurgical ; l'association des deux est indispensable dans tous les cas. En effet, en l'absence de traitement chirurgical, le traitement médical seul est voué à l'échec dans 95 à 100 % des cas. La chirurgie élective reste la pierre angulaire du traitement des anévrysmes mycotiques de l'AMS [1], néanmoins, l'anévrisme mycotique cérébral peut parfaitement disparaitre sous une antibiothérapie bien conduite [7] comme ce qui a été noté chez notre patient.

## Conclusion

L'endocardite infectieuse est une pathologie peu fréquente grevée d'un pronostic péjoratif du fait de ses complications, notamment les anévrismes artériels mycotiques dont la double localisation cerebro-mésentérique est une urgence diagnostique et thérapeutique rare, mais à lourde morbi-mortalité. En pratique, l'angioscanner est l'examen de diagnostic de ces complications. Le traitement est obligatoirement médico-chirurgical pour la localisation viscérale et peut parfois se contenter d'antibiothérapie bien menée pour les anévrismes mycotiques cérébrales.
